# Downregulation of hsa_circ_0007580 inhibits non-small cell lung cancer tumorigenesis by reducing miR-545-3p sponging

**DOI:** 10.18632/aging.103472

**Published:** 2020-07-18

**Authors:** Shuifang Chen, Shan Lu, Yinan Yao, Junjun Chen, Guangdie Yang, Lingfang Tu, Zeying Zhang, Jianli Zhang, Lina Chen

**Affiliations:** 1 The First Affiliated Hospital, College of Medicine, Zhejiang University, Hangzhou 310003, China

**Keywords:** NSCLC, hsa_circ_0007580, miR-545-3p, PRKCA

## Abstract

Non-small cell lung cancer (NSCLC) is a highly malignant tumor. Many circular RNAs (circRNAs) are reportedly in regulating the progression of NSCLC. To identify potential therapeutic targets for NSCLC, we conducted a bioinformatics analysis of circRNAs differentially expressed between NSCLC tissues and adjacent normal tissues. Hsa_circ_0007580 was upregulated in NSCLC tumor tissues, and the expression of its host gene (protein kinase Ca) correlated negatively with overall survival. Short-hairpin RNAs were used to knock down hsa_circ_0007580 in NSCLC cells, and gene and protein levels were measured with qRT-PCR and Western blotting, respectively. NSCLC cell proliferation, migration and apoptosis were evaluated with CCK-8 assays, Ki-67 staining, Transwell assays and flow cytometry, respectively. Knocking down hsa_circ_0007580 inhibited proliferation and invasion by NSCLC cells and induced their apoptosis. Dual luciferase reporter assays indicated that miR-545-3p can bind to hsa_circ_0007580 (suggesting that hsa_circ_0007580 sponges miR-545-3p) and to protein kinase Ca (suggesting that miR-545-3p directly inhibits this gene). In a xenograft tumor model, downregulating hsa_circ_0007580 inhibited NSCLC tumorigenesis by inactivating p38/mitogen-activated protein kinase signaling. Thus, silencing hsa_circ_0007580 notably inhibited NSCLC progression *in vitro* and *in vivo*, suggesting this circRNA could be a novel treatment target for NSCLC.

## INTRODUCTION

Lung cancer is the most commonly diagnosed cancer and the leading cause of cancer death globally [1]. Lung cancers can be divided into non-small cell lung cancer (NSCLC) and small cell lung cancer. Approximately 83% of all lung cancers are NSCLC, and about 80% of patients with NSCLC are diagnosed in the advanced stages [2]. Much effort has been made to treat NSCLC, but the prognosis remains grim [3]. Thus, it is urgent to find a new strategy for treating NSCLC.

Circular RNAs (circRNAs) are endogenous RNAs characterized by a covalently closed cyclic structure [4]. Intracellular circRNAs with competing endogenous RNA activity may function as sponges for microRNAs (miRNAs) because they contain miRNA response elements. This greatly inhibits miRNA activity and ultimately upregulates miRNA target genes [5, 6]. Therefore, circRNAs are important biological regulators that should be explored for both their contribution to disease mechanisms and their potential as therapeutic targets.

Previous reports have indicated that circRNAs can alter gene expression in cancer-associated signaling pathways [7, 8]. Moreover, circRNAs may be dysregulated in malignant tumors and contribute to the tumorigenesis of many cancer types [9, 10]. However, the function of circRNAs during the progression of NSCLC remains unclear. In this study, we performed a bioinformatics analysis to identify circRNAs that were differentially expressed in NSCLC tissues, in order to determine potential treatment targets for this disease.

## RESULTS

### Expression profiles of circRNAs in NSCLC

**Figure 1:**
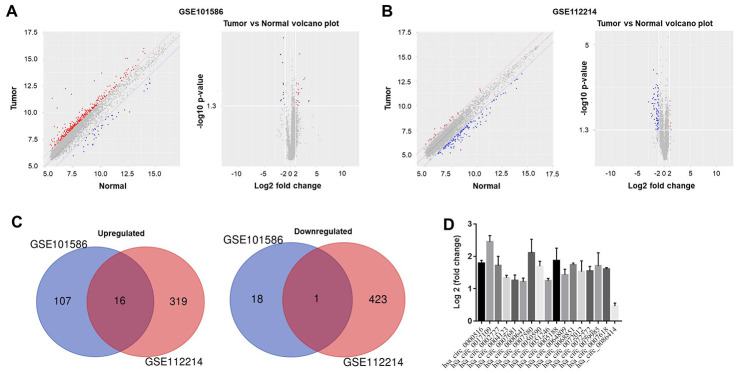
**Differentially expressed circRNAs in NSCLC.** The differentially expressed circRNAs between NSCLC tissues and adjacent normal tissues in (**A**) GSE101586 or (**B**) GSE112214 were assessed using principal component analysis and a volcano plot. Red indicates high expression while blue indicates low expression. (**C**) Among the differentially expressed circRNAs in GSE101586, 18 were downregulated and 107 were upregulated in NSCLC tissues. In GSE112214, 423 circRNAs were downregulated and 319 were upregulated in NSCLC tissues. (**D**) The overlapping differentially expressed circRNAs between GSE101586 and GSE112214 were analyzed.

To analyze the differentially expressed circRNAs in NSCLC, we performed a bioinformatics analysis of NSCLC tissues and adjacent normal tissues in the GSE101586 and GSE112214 data sets. The results were evaluated using principal component analysis and a volcano plot (Figure 1A and 1B). Among the differentially expressed circRNAs in GSE101586, 18 were downregulated and 107 were upregulated in NSCLC tissues compared with adjacent normal tissues. In GSE112214, 423 circRNAs were downregulated and 319 were upregulated in NSCLC tissues (Figure 1C). The overlap of these differentially expressed circRNAs between the two data sets is presented in Figure 1D.

**Figure 2:**
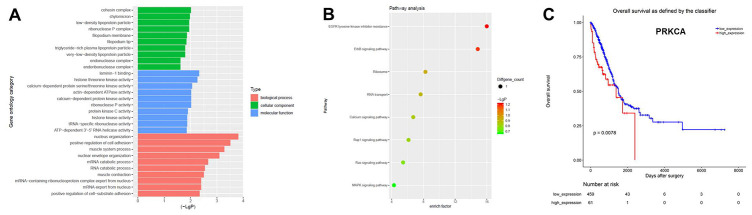
**CircRNA expression profiles in NSCLC analyzed with GO and pathway analyses.** (**A**) GO analysis was used to explore the potential functions of the differentially expressed circRNAs. (**B**) Pathway analysis was used to explore the signaling pathways associated with the host genes of the overlapping circRNAs. (**C**) The Cancer Genome Atlas was used to identify circRNA host genes associated with the prognosis of NSCLC.

Next, Gene Ontology (GO) and pathway analyses were performed based on the host genes of the circRNAs. As shown in Figure 2A, the most common biological process among the host genes was nucleus organization, while the most enriched cellular component was the cohesion complex and the most enriched molecular function was laminin-1 binding. Pathway analysis revealed that the host genes of the overlapping circRNAs were associated with the mitogen-activated protein kinase (MAPK) signaling pathway (Figure 2B). The Cancer Genome Atlas database indicated that one of the host genes, *PRKCA* (encoding protein kinase Cα), was associated with the prognosis of NSCLC (Figure 2C). Based on these results, hsa_circ_0007580 (the circRNA corresponding to this host gene) was selected for subsequent experiments.

### Downregulation of hsa_circ_0007580 significantly inhibited the proliferation of NSCLC cells

**Figure 3:**
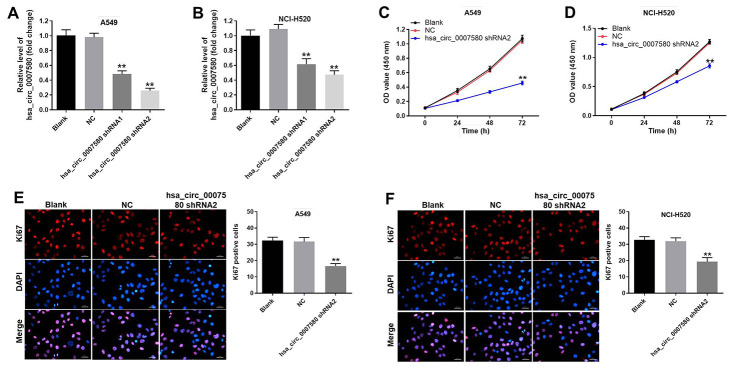
**Downregulation of hsa_circ_0007580 significantly inhibited NSCLC cell proliferation.** NSCLC cells were transfected with hsa_circ_0007580 shRNA1 or shRNA2 for 24 h. (**A**) The expression of hsa_circ_0007580 in A549 cells was detected using qRT-PCR. (**B**) The expression of hsa_circ_0007580 in NCI-H520 cells was detected using qRT-PCR. The optical density value of (**C**) A549 or (**D**) NCI-H520 cells was assessed following the CCK-8 assay. The proliferation of (**E**) A549 or (**F**) NCI-H520 cells was tested by Ki-67 staining. Red fluorescence indicates Ki-67. Blue fluorescence indicates DAPI; 200× magnification. ^**^P<0.01 vs. control.

Next, we used short-hairpin RNAs (shRNAs) to knock down hsa_circ_0007580 in NSCLC cells. The transfection efficiency was assessed using quantitative real-time PCR (qRT-PCR), which indicated that shRNA1 and shRNA2 each significantly downregulated hsa_circ_0007580 expression in NSCLC cells (Figure 3A and 3B). These data suggested that the hsa_circ_0007580 shRNAs were stably transfected into A549 and NCI-H520 cells. Since hsa_circ_0007580 shRNA2 exhibited a better transfection efficiency, it was used for subsequent experiments.

Next, a Cell Counting Kit 8 (CCK-8) assay was performed to detect cell viability. The results demonstrated that silencing of hsa_circ_0007580 notably inhibited NSCLC cell viability (Figure 3C and 3D). Moreover, the data of Ki-67 staining revealed that knockdown of hsa_circ_0007580 significantly suppressed NSCLC cell proliferation (Figure 3E and 3F).

### Hsa_circ_0007580 shRNA2 notably induced the apoptosis and inhibited the invasion of NSCLC cells

**Figure 4:**
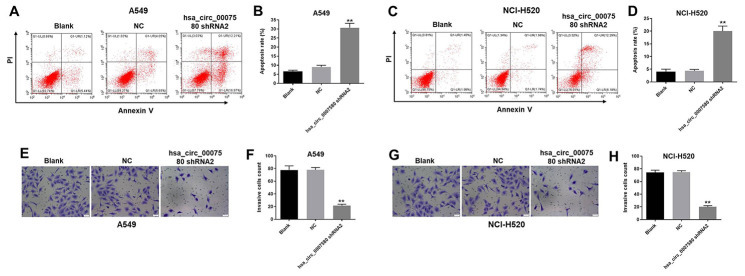
**Hsa_circ_0007580 shRNA2 notably induced the apoptosis and inhibited the invasion of NSCLC cells.** (**A**–**D**) The proportion of apoptotic cells was detected using fluorescence-activated cell sorting after double staining with Annexin V and PI. X axis: the level of Annexin-V fluorescein isothiocyanate fluorescence; Y axis: the PI fluorescence. (**E**–**H**) The invasion of NSCLC cells was tested with a Transwell invasion assay; 400× magnification. ^**^P<0.01 vs. control.

Next, flow cytometry was used to investigate the effects of hsa_circ_0007580 shRNA on cell apoptosis. As shown in Figure 4A–4D, hsa_circ_0007580 shRNA2 clearly induced NSCLC cell apoptosis. Additionally, NSCLC cell invasion was markedly inhibited when the cells were treated with hsa_circ_0007580 shRNA2 (Figure 4E–4H). A549 cells were more sensitive than NCI-H520 cells to hsa_circ_0007580 shRNA2, so A549 cells were used in subsequent experiments. Altogether, these data suggested that the silencing of hsa_circ_0007580 induced the apoptosis and inhibited the invasion of NSCLC cells.

### MiR-545-3p was the downstream target of hsa_circ_0007580

**Figure 5:**
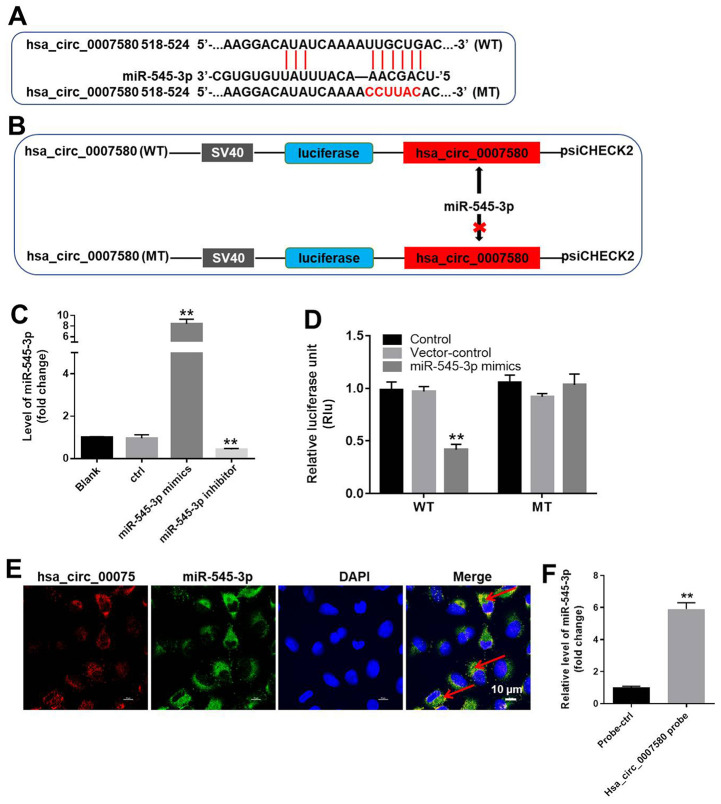
**MiR-545-3p was the downstream target of hsa_circ_0007580.** (**A**, **B**) The gene structure of hsa_circ_0007580 at positions 518-524 contained the predicted target site of miR-545-3p, with a sequence of CCUUAC. (**C**) The expression of miR-545-3p in A549 cells was detected using qRT-PCR. (**D**) Luciferase activity was measured with a dual luciferase reporter assay in A549 cells co-transfected with the WT/MT hsa_circ_0007580 plasmid and miR-545-3p. (**E**) The colocalization of hsa_circ_0007580 and miR-545-3p was detected using FISH. (**F**) RNA pull-down assay was performed to confirm the correlation between hsa_circ_0007580 and miR-545-3p. ^**^P<0.01 vs. control.

To investigate the mechanism by which hsa_circ_0007580 induced the progression of NSCLC, we analyzed the CircInteractome (https://circinteractome.nia.nih.gov/). As indicated in Figure 5A and 5B, miR-545-3p was detected as a possible downstream target of hsa_circ_0007580. We then transfected NSCLC cells with miR-545-3p mimics or inhibitors, and performed qRT-PCR to verify the transfection efficiency. The expression of miR-545-3p was notably upregulated by miR-545-3p mimics but downregulated by miR-545-3p inhibitors (Figure 5C).

Next, we performed a dual luciferase reporter assay to determine whether miR-545-3p could bind to hsa_circ_0007580. Indeed, miR-545-3p mimics reduced the luciferase activity of a wild-type (WT) hsa_circ_0007580 reporter, but not a mutated (MT) reporter sequence (Figure 5D). Fluorescence in situ hybridization (FISH) confirmed that miR-545-3p co-localized with hsa_circ_0007580 in NSCLC cells (Figure 5E). Furthermore, the data of RNA pull-down demonstrated that hsa_circ_0007580 could bind to miR-545-3p (Figure 5F). Given the notion that circRNAs function as miRNA sponges, these data suggested that miR-545-3p is a downstream target of hsa_circ_0007580.

### *PRKCA* was the direct target of miR-545-3p

**Figure 6:**
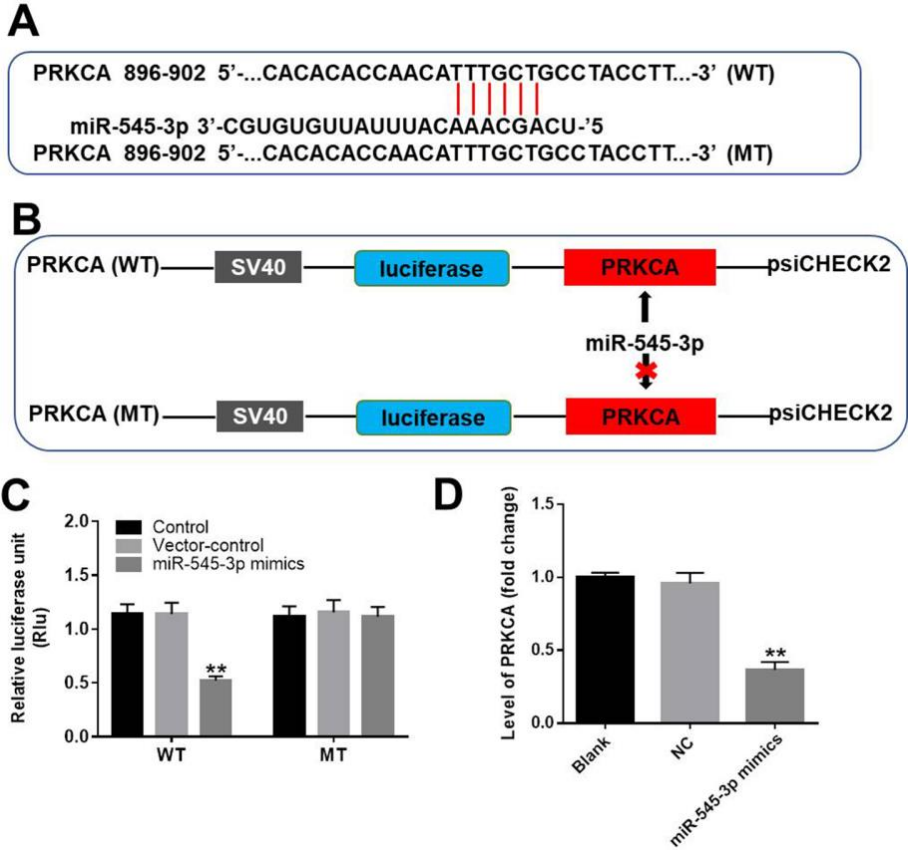
***PRKCA* was a direct target of miR-545-3p.** (**A**, **B**) The gene structure of *PRKCA* at positions 896-902 contained the predicted target site of miR-545-3p in its 3'-UTR, with a sequence of TTTGCT. (**C**) Luciferase activity was measured with a dual luciferase reporter assay in A549 cells co-transfected with the WT/MT *PRKCA* 3′-UTR plasmid and miR-545-3p. (**D**) The expression of *PRKCA* in NSCLC cells was investigated using qRT-PCR. ^**^P<0.01 vs. control.

We next used TargetScan (http://www.targetscan.org/vert_71/) and the miRDB (http://www.mirdb.org/) to search for miR-545-3p target genes, and we verified the results using a dual luciferase reporter assay. As demonstrated in Figure 6A–6C, *PRKCA* was identified as a direct target of miR-545-3p. We then used qRT-PCR to assess *PRKCA* expression in NSCLC cells transfected with miR-545-3p mimics. As shown in Figure 6D, *PRKCA* expression was notably downregulated in NSCLC cells overexpressing miR-545-3p. These results indicated that miR-545-3p directly inhibited *PRKCA* expression.

### Hsa_circ_0007580 silencing inhibited the progression of NSCLC by inactivating MAPK signaling

**Figure 7:**
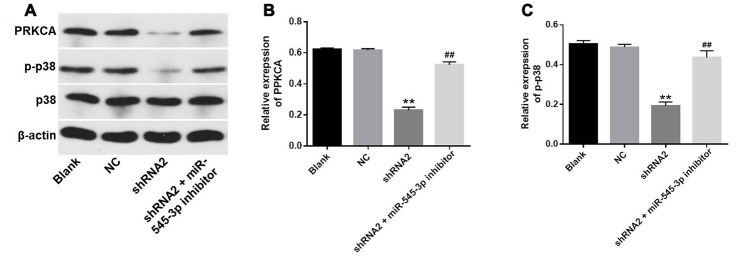
**Hsa_circ_0007580 silencing inhibited the progression of NSCLC by inactivating MAPK signaling.** (**A**) The protein levels of PRKCA, p38 and p-p38 in A549 cells were investigated through Western blotting. (**B**) The relative expression of PRKCA was quantified and normalized to that of β-actin. (**C**) The relative expression of p-p38 in A549 cells was quantified and normalized to that of β-actin. ^**^P<0.01 vs. control.

To further explore the mechanism by which downregulating hsa_circ_0007580 suppressed the progression of NSCLC, we transfected NSCLC cells with hsa_circ_0007580 shRNA with or without miR-545-3p inhibitors. Western blotting revealed that the protein levels of phosphorylated (p)-p38 and PRKCA were significantly downregulated in NSCLC cells treated with hsa_circ_0007580 shRNA, while these results were partially reversed in the presence of miR-545-3p inhibitors (Figure 7A–7C). These data demonstrated that the silencing of hsa_circ_0007580 inhibited the progression of NSCLC by inactivating MAPK signaling.

### Hsa_circ_0007580 shRNA2 significantly inhibited NSCLC tumor growth *in vivo*

**Figure 8:**
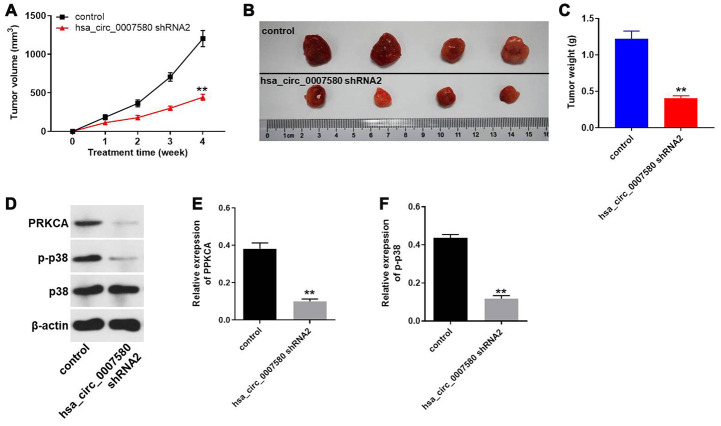
**Hsa_circ_0007580 shRNA2 significantly attenuated the symptoms of NSCLC *in vivo*.** Mice were injected with the vector-control or hsa_circ_0007580 shRNA2. (**A**) The tumor volumes of the mice were measured weekly for four weeks. (**B**) At the end of the study, the tumor tissues were collected and photographed. (**C**) The tumor weights in each group of mice were calculated. (**D**) The protein levels of PRKCA, p38 and p-p38 in tumor tissues from the mice were detected through Western blotting. (**E**) The relative expression of PRKCA was quantified and normalized to that of β-actin. (**F**) The relative expression of p-p38 was quantified and normalized to that of β-actin. ^**^P<0.01 vs. control.

Finally, a xenograft mouse model was established to detect the effects of hsa_circ_0007580 shRNA on NSCLC *in vivo*. The tumor sizes (Figure 8A and 8B) and tumor weights (Figure 8C) of the mice were significantly reduced when hsa_circ_0007580 was knocked down. Additionally, Western blotting indicated that PRKCA and p-p38 protein levels in tumor tissues were significantly reduced in mice treated with hsa_circ_0007580 shRNA (Figure 8D–8F). These results demonstrated that silencing hsa_circ_0007580 significantly attenuated the symptoms of NSCLC *in vivo*.

## DISCUSSION

CircRNA, a type of noncoding RNA, differs from traditional linear RNA, and is widely distributed in mice and humans [11–13]. It has been reported that circRNAs can up- or downregulate gene expression and even encode proteins [14]. CircRNAs are stable and widely expressed in many tumor tissues [15]. Due to their important biological functions, some circRNAs can be used as biomarkers for the diagnosis of diseases [16]. It is possible that circRNAs, like miRNAs, are also involved in paracrine signaling or cell-to-cell crosstalk. In this study, we found that hsa_circ_0007580 promoted the tumorigenesis of NSCLC. Knocking down hsa_circ_0007580 inhibited the proliferation and induced the apoptosis of NSCLC cells. Many studies have indicated that circRNAs regulate the progression of NSCLC [17–19], but our research is the first to demonstrate the biological function of hsa_circ_0007580 in NSCLC. However, Pang W et al found that hsa_circ_0072309 inhibits the tumorigenesis of NSCLC [20]. This discrepancy may due to different circRNA function.

MiRNAs are key contributors to the development of multiple diseases, including NSCLC [21, 22]. In this research, we found that downregulating miR-545-3p partially reversed the inhibitory effects of hsa_circ_0007580 knockdown on NSCLC progression. Changjun L et al. found that miR-545-3p could downregulate the cell proliferation, invasion and migration in NSCLC [23]. Similarly, our findings indicated that miR-545-3p could be a key inhibitor of NSCLC progression. In addition, Lu et al. demonstrated that the circRNA HIPK3 could induce cell proliferation and inhibit apoptosis in NSCLC by sponging miR-149 [17]. Our data revealed a similar mechanism, as downregulating hsa_circ_0007580 inhibited NSCLC tumorigenesis by reducing the sponging of miR-545-3p.

PRKCA is a member of the protein kinase C family. The upregulation of PRKCA has been observed in multiple malignant tumors and found to induce cancer cell proliferation and metastasis [24–26]. We found that *PRKCA* was a direct target of miR-545-3p. A previous report indicated that miR-216a induced the apoptosis of breast cancer cells by downregulating *PRKCA* [27]. Moreover, PRKCA has been confirmed to induce autophagy in lung cancer cell lines [28]. Our results were consistent with these findings, suggesting that PRKCA may promote the development of NSCLC.

According to Qiu et al [29], circPRKCI could sponge miR-545 in lung cancer. Our research was similar to this previous finding. The host genes of circPRKCI (PRKCI) and hsa_circ_0007580 (PRKCA) are members of Protein Kinase C (PKC) family [30]. In addition, PKC family has been proved to be closely correlated with prognosis of lung cancer [31, 32]. This background may contribute to the similar function between hsa_circ_0007580 and circPRKCI. Meanwhile, Qiu et al found that circPRKCI could regulate E2F7/Cyclin D1. Indeed, PRKCA is known to mediate G2/M phase arrest [33]. However, E2F7 has been confirmed to induce G1 phase arrest [34]. Therefore, the different mechanisms of hsa_circ_0007580 and circPRKCI in lung cancer may due to different functions of E2F7 and PRKCA.

P38 is expressed in a many cell types, and can promote the progression of cancer by activating MAPK [35, 36]. In the present study, knockdown of hsa_circ_0007580 significantly inactivated p38/MAPK signaling. A previous report indicated that the activation of p38 MAPK could contribute to the stem cell-like properties of NSCLC [37]. Additionally, it has been proved that PRKCA can activate p38/MAPK signaling in NSCLC [38]. Our research was consistent with these findings, suggesting that p38/MAPK could promote the progression of NSCLC. Frankly speaking, this study focused only on p38/MAPK signaling so far. Since phosphoinositide 3-kinase/Akt signaling has also been reported to be involved in the development of NSCLC [39], we will further investigate the effects of hsa_circ_0007580 on phosphoinositide 3-kinase/Akt signaling.

In conclusion, the downregulation of hsa_circ_0007580 inhibited the tumorigenesis of NSCLC by reducing the sponging of miR-545-3p; thus, this circRNA could be a new target for the treatment of NSCLC.

## MATERIALS AND METHODS

### Cell culture

A549, NCI-H520 and 293T cell lines were obtained from the American Type Culture Collection (Manassas, VA, USA) and cultured in Dulbecco’s Modified Eagle’s Medium (DMEM, Thermo Fisher Scientific, Waltham, MA, USA) with 10% fetal bovine serum (Thermo Fischer Scientific), 1% penicillin and streptomycin (Thermo Fisher Scientific) at 37°C and 5% CO_2._

### Bioinformatics analysis

Two datasets (GSE101586 and GSE112214) containing gene expression data for NSCLC and adjacent normal tissues (controls) were obtained from the Gene Expression Omnibus database (https://www.ncbi.nlm.nih.gov/geo/). Principal component analysis and volcano plot were performed to asses the expressions of cirRNAs in NSCLC and adjacent normal tissues. GO analysis was performed to explore the functions of circRNA host genes in terms of biological processes, cellular components and molecular functions. Biological pathways were assessed in the Kyoto Encyclopedia of Genes and Genomes. Survival curves were generated using The Cancer Genome Atlas (https://www.cancer.gov/about-nci/organization/ccg/research/structural-genomics/tcga).

### qRT-PCR

Total RNA from NSCLC cell lines was extracted with TRIzol reagent (TaKaRa, Tokyo, Japan) according to the manufacturer’s protocol. Then, cDNA was synthesized with a reverse transcription kit (TaKaRa, Ver. 3.0) according to the manufacturer’s protocol. The following protocol was used to perform qRT-PCR in triplicate: 2 minutes at 94°C, followed by 35 cycles of 30 seconds at 94°C and 45 seconds at 55°C. The following primers were obtained from GenePharma (Shanghai, China): Hsa_circ_0018818: forward 5’-CAGGACCTTCTGTGGGACTC-3’ and reverse 5’-TCCAAAACTCCCCTTTCCCA-3’. MiR-545-3p: forward 5’- TGCGCTCAGCAAACATTTATTG-3’ and reverse 5’- CCAGTGCAGGGTCCGAGGTATT-3’. β-actin: forward 5’-AGCGAGCATCCCCCAAAGTT-3’ and reverse 5’-GGGCACGAAGGCTCATCATT-3’. U6: forward 5’-CGCTTCGGCAGCACATATAC-3’ and reverse 5’- AAATATGGAACGCTTCACGA-3’. The relative fold changes were calculated with the 2^-ΔΔCt^ method using the formula: 2^-(sample ΔCt – control ΔCt)^, where ΔCt is the difference between the fluorescent amplification thresholds of the gene of interest and the internal reference gene used for normalization (U6 or β-actin).

### Cell transfection

Two shRNAs directly targeting hsa_circ_0007580 (shRNA1 and shRNA2) and one shRNA with a nontargeting sequence (negative control) were obtained from Hanbio Biotechnology Co., Ltd (Shanghai, China) and packaged into lentiviruses. The lentiviral vector DNAs were then transfected into 293T cells, and the cells were incubated at 32°C. Then, the supernatants were collected and filtered for the retrieval of lentiviral particles. Finally, NSCLC cells were infected with the lentiviral particles according to the manufacturer’s protocol. After 48 h of incubation, stably transfected NSCLC cells were selected with puromycin (2.5 μg/mL, Sigma Aldrich, St. Louis, MO, USA), and qRT-PCR was used to verify the efficiency of transfection.

For miR-545-3p transfection, Lipofectamine 2000 was used to transfect A549 or NCI-H520 cells with miR-545-3p mimics, miR-545-3p inhibitors or negative controls, as described previously [40]. The mimic, inhibitor and negative control RNAs were purchased from GenePharma (Shanghai, China). The efficiency of transfection was detected with qRT-PCR.

### CCK-8 assay

A549 or NCI-H520 cells were seeded in 96-well plates (5×10^3^ cells per well) overnight. Then, the cells were treated with hsa_circ_0007580 shRNA2 or the negative control for 0, 24, 48 or 72 h. The cells in each well were then treated with 10 μL of CCK-8 reagent and further incubated for 2 h at 37°C. Finally, the absorbance of the NSCLC cells was measured at 450 nm on a microplate reader (Thermo Fisher Scientific).

### Ki-67 staining

NSCLC cells were seeded in 24-well plates overnight. Next, cells were treated with negative control or hsa_circ_0007580 shRNA2 for 72 h. Then, cells were blocked with 10% goat serum for 30 min at room temperature and then incubated with anti-Ki67 antibody (Abcam, Cambridge, MA, USA; 1:1000) at 4°C overnight, After that, cells were incubated with goat anti-rabbit IgG (Abcam; 1:5000) at 37°C for 1 h. The nuclei were stained with DAPI (Beyotime, Shanghai, China) for 5 min. Finally, cells were observed under a fluorescence microscope (Olympus CX23, Tokyo, Japan).

### Cell apoptosis analysis

A549 or NCI-H520 cells were trypsinized, washed with phosphate-buffered saline and resuspended in Annexin V Binding Buffer. The cells were then stained with 5 μL of fluorescein isothiocyanate and 5 μL propidium iodide (PI) for 15 minutes. A flow cytometer (BD, Franklin Lakes, NJ, USA) was used to determine the cell apoptosis rate.

### Dual luciferase reporter assay

For the construction of the WT/MT reporter vectors for hsa_circ_0007580 and *PRKCA,* respectively, the partial sequences of hsa_circ_0007580 and the 3′-untranslated region (UTR) of *PRKCA* containing the putative binding sites for miR-545-3p were synthesized by Sangon Biotech (Shanghai, China) and cloned into pmirGLO Dual-Luciferase miRNA Target Expression Vectors (Promega, Madison, WI, USA). Lipofectamine 2000 (Thermo Fisher Scientific) was used to transfect 293T cells with the hsa_circ_0007580/*PRKCA* (WT) or hsa_circ_0007580/*PRKCA* (MT) vectors, together with the control, vector-control or miR-545-3p mimics, according to the manufacturer’s instructions. The relative luciferase activity was analyzed on a Dual-Glo Luciferase Assay System (Promega).

### RNA pull-down

For RNA pull-down assay, the Biotin RNA Labeling Mix (Roche, Basel, Switzerland) was used to transcribe and label probe-control or probe-hsa_circ_0007580 from hsa_circ_0007580 shRNA2 lenti vector *in vitro*. An RNA structure buffer (Thermo Fisher Scientific) was used to induce secondary structure formation from the biotin-labeled RNAs. Streptavidin beads (Thermo Fisher Scientific) were washed three times with 500 μL of RNA immunoprecipitation wash buffer (Thermo Fisher Scientific) and then added to the biotinylated RNAs at 4°C overnight. The overnight mixture was separated by a magnetic field so that streptavidin bead-RNA complexes could be obtained. Then, lysates of NSCLC cells were added to the complexes and incubated on a rotator at room temperature for one hour. The incubated mixture was again separated with a magnetic field so that streptavidin bead-RNA-protein complexes could be obtained.

### FISH detection

The co-localization of miR-545-3p and hsa_circ_0007580 in the cytoplasm was investigated using FISH detection as described previously [41].

### Western blotting

Total protein was isolated from cell lysates or tumor tissues with radio-immunoprecipitation assay buffer and quantified with a bicinchoninic acid protein assay kit (Beyotime, Shanghai, China). Proteins were resolved on 10% sodium dodecyl sulfate polyacrylamide gels and then transferred to polyvinylidene difluoride membranes (Bio-Rad). After being blocked, the membranes were incubated with primary antibodies at 4°C overnight and then incubated with an anti-rabbit secondary antibody (Abcam; 1:5000) at room temperature for 1 h. The membranes were scanned on an Odyssey Imaging System and analyzed with Odyssey v2.0 software (LICOR Biosciences, Lincoln, NE, USA). The primary antibodies used in this study were: anti-p38 (Abcam, Cambridge, MA, USA; 1:1000), anti-PRKCA (Abcam; 1:1000) and anti-β-actin (Abcam; 1:1000). β-actin was used as an internal control.

### In vivo study

Eight BALB/c nude mice (six to eight weeks old) were purchased from Vital River (Beijing, China). The mice were housed in a dedicated specific-pathogen-free facility. A549 cells (control or stably expressing hsa_circ_0007580 shRNA2) were transplanted subcutaneously into each mouse as described previously [42]. The tumor volume was measured weekly as previously reported [43]. At the end of the experiment, the mice were sacrificed and their tumors were collected and weighed. All *in vivo* experiments were performed in accordance with the National Institutes of Health Guide for the Care and Use of Laboratory Animals, following a protocol approved by the Ethics Committees of The First Affiliated Hospital, Zhejiang University.

### Statistical analysis

For each analysis, at least three independent experiments were performed. All data are expressed as the mean ± standard deviation. Differences were analyzed with Student’s t-test (for two groups) or one-way analysis of variance followed by Tukey’s test (for three or more groups) in GraphPad Prism 7. P<0.05 was considered to indicate a statistically significant difference.
